# Differences in K-*ras* and mitochondrial DNA mutations and microsatellite instability between colorectal cancers of Vietnamese and Japanese patients

**DOI:** 10.1186/s12876-014-0203-0

**Published:** 2014-11-30

**Authors:** Tomohiro Miwata, Toru Hiyama, Duc Trong Quach, Huy Minh Le, Ha Ngoc Thi Hua, Shiro Oka, Shinji Tanaka, Koji Arihiro, Kazuaki Chayama

**Affiliations:** Department of Gastroenterology and Metabolism, Graduate School of Biomedical Sciences, Hiroshima University, Hiroshima, Japan; Health Service Center, Hiroshima University, Higashihiroshima, Japan; Department of Endoscopy, University Medical Center, Ho Chi Minh, Vietnam; Department of Pathology, University Medical Center, Ho Chi Minh, Vietnam; Department of Endoscopy, Hiroshima University Hospital, Hiroshima, Japan; Department of Pathology, Hiroshima University Hospital, Hiroshima, Japan

**Keywords:** Colorectal cancer, Vietnamese, Japanese, K-*ras*, Mitochondrial DNA, Microsatellite instability, D310, BAT-26, Mutation

## Abstract

**Background:**

The incidence of early-onset (under 50 years of age) colorectal cancer (CRC) in the Vietnamese has been reported to be quite higher than that in the Japanese. To clarify the differences in genetic alterations between Vietnamese and Japanese CRCs, we investigated mutations in K-*ras* and mitochondrial DNA (mtDNA) and high-frequency microsatellite instability (MSI-H) in the CRCs of Vietnamese and Japanese patients.

**Methods:**

We enrolled 60 Vietnamese and 233 Japanese patients with invasive CRCs. DNA was extracted from formalin-fixed, paraffin-embedded tissue sections. K-*ras* mutations were examined with PCR-single-strand conformation polymorphism analysis. mtDNA mutations and MSI-H were examined with microsatellite analysis using D310 and BAT-26, respectively.

**Results:**

K-*ras* mutations were examined in 60 Vietnamese and 45 Japanese CRCs. The frequency of the mutations in the Vietnamese CRCs was significantly higher than that in the Japanese CRCs (8 of 24 [33%] vs 5 of 45 [11%], *p* =0.048). MSI-H was examined in 60 Vietnamese and 130 Japanese CRCs. The frequency of MSI-H in the Vietnamese CRCs was also significantly higher than that in the Japanese CRCs (6 of 27 [22%] vs 10 of 130 [8%], *p* =0.030). mtDNA mutations were examined in 60 Vietnamese and 138 Japanese CRCs. The frequency of mtDNA mutations in the Vietnamese CRCs was significantly higher than that in the Japanese CRCs (19 of 44 [43%] vs 11 of 133 [9%], *p* <0.001). There were no significant differences in clinicopathologic characteristics, such as age, sex, tumour location, and depth, in terms of tumours with/without each genetic alteration in the CRCs of the Vietnamese and Japanese patients.

**Conclusions:**

These results indicate that the developmental pathways of CRCs in the Vietnamese may differ from those of CRCs in the Japanese.

## Background

Colorectal cancer (CRC) is one of the most common cancers in the world. Especially, the incidences and mortality rates of CRCs in developing and economically transitioning countries are rapidly rising [1]. Vietnam is one of the developing countries in Asia, and Japan is one of the developed countries. In Vietnam, CRC is the third most common cause of cancer deaths in men and fourth in women [2]. In addition, a high incidence of early-onset CRC (that occurring in patients under 50 years of age) has been reported: the ratio of early-onset CRC to all CRCs is approximately 28% [2]. On the other hand, CRC is the third most common cause of cancer death in men and first in women in Japan. The ratio of early-onset CRC to all CRCs is reported to be approximately 6% [3]. Thus, the incidence of early-onset CRCs in the Vietnamese may be quite higher than that in the Japanese, implying that the pathways of CRC development, *i.e*. the pattern of genetic changes in CRCs, in the Vietnamese may differ from those in the Japanese.

To date, at least 3 distinct pathways of CRC development are known [4-6]. One is the chromosomal instability pathway, which is present in 65–70% of CRCs. It is associated with the activation of oncogenes, such as K-*ras* mutations, and the inactivation of tumour suppressor genes such *p53* and *DCC*. Second is the microsatellite instability (MSI) pathway, which is present in approximately 15% of CRCs. Third is the mitochondrial DNA (mtDNA) dysfunction pathway, which is present in approximately 5% of CRCs. However, there are few studies on the genetic alterations in CRCs in the Vietnamese. Therefore, we investigated mutations of K-*ras* and mtDNA and high-frequency MSI (MSI-H), the representative genetic alterations of the 3 pathways, in the CRCs of Vietnamese and Japanese patients to clarify the differences between CRCs in these two populations.

## Methods

### Patients

We enrolled 60 patients with invasive CRCs diagnosed at the Department of Endoscopy, University Medical Center in Ho Chi Minh, Vietnam, between March 2009 and March 2011, and 233 patients with invasive CRCs diagnosed at the Department of Endoscopy, Hiroshima University Hospital, Hiroshima, Japan, between 1998 and 2010. The patients’ clinicopathologic data were retrieved from the databases of both hospitals. This retrospective study was approved by the ethical committees of both the University Medical Center in Ho Chi Minh and Hiroshima University. Written informed consent was obtained from the patient for the publication of this report and any accompanying images.

### DNA extraction and amplification

Formalin-fixed, paraffin-embedded tissue sections of 10 μm in thickness were stained with haematoxylin and eosin, dehydrated in graded ethanol, and then dried without a cover glass. Tissues were cut with sterile scissors, and the DNA was extracted from the tissues with 20 μl of extraction buffer (100 mM Tris–HCl; 2 mM ethylene diamine tetraacetic acid, pH 8.0; and 400 μg/ml proteinase K) at 55°C for 3 h. The tubes were boiled for 7 min to inactivate proteinase K, and then 1–5 μl of these extracts was used for each polymerase chain reaction (PCR) amplification [7].

### Mutational screening and direct sequencing of K-*ras*

DNA samples were screened for mutations of K-*ras* by PCR-single-strand conformation polymorphism (SSCP) analysis. The PCR primers were designed to amplify mutation hot spots, codons 12 and 13, of the K-*ras* [8]. PCR-SSCP analysis was performed as described previously [8]. Briefly, each 25-_μ_L reaction mixture contained 1 × AmpliTaq Gold Buffer (8.0 mmol/L Tris–HCl, pH 8.3; 40 mmol/L KCl; Perkin-Elmer, Branchburg, NJ, USA), 4 mmol/L MgCl_2_, 0.3 mmol/L of each deoxynucleotide triphosphate, 100 pmol of each primer, 10–20 ng genomic DNA, 2.5 mCi [_α_^32^-P]dCTP (3000 Ci/mmol/L, 10 mCi/mL), and 1.25 U AmpliTaq Gold DNA polymerase (Perkin-Elmer). The reaction mixtures were heated to 95°C for 10 min, followed by 45 cycles of denaturation at 94°C for 1 min, annealing at 55°C for 2 min, and strand elongation at 72°C for 2 min. After PCR, the samples were electrophoresed on 6% polyacrylamide gels (ratio of acrylamide:bis-acrylamide, 19:1) with 10% glycerol at 4°C. The gels were then subjected to autoradiography overnight at −80°C on Fuji RX film (Fuji Film, Minamiashigara, Japan).

### Microsatellite assay

A microsatellite marker, BAT-26, was used to examine MSI-H [9]. The microsatellite assay was performed as described previously [10]. Briefly, each 15-μL reaction mixture containing 10–20 ng of genomic DNA, 6.7 mmol Tris–HCl (pH 8.8), 6.7 mmol EDTA, 6.7 mmol MgCl_2_, 0.33 μmol primer labelled with (gamma-^32^P) dATP, 0.175 μmol unlabelled primer, 1.5 mmol of each deoxynucleotide triphosphate, and 0.75 units of AmpliTaq Gold DNA polymerase was amplified for 40 cycles as follows: denaturation at 94°C for 30 s, annealing at 55°C for 30 s, and strand elongation at 72°C for 30 s. The PCR products were electrophoresed on 6% polyacrylamide, 8 mol urea, and 32% formamide gels and subjected to autoradiography overnight at −80°C on Fuji RX film.

### Mutational analysis of mtDNA

A 109-bp fragment containing the D310 repeat of mtDNA, the D-loop region, was amplified. The primer sequences included 5-ACAATT’GAATGTCTGCACAGCCACTT-3’ for the sense primer and 5’-GGCAGAGATGTGTTTAAGTGCTG-3’ for the antisense primer [5]. The microsatellite assays were carried out to analyse the mutation of the D310 repeat of mtDNA.

### Statistical analysis

We compared the frequency of genetic abnormalities between the CRCs of the Vietnamese and Japanese patients. Statistical differences were evaluated using the Student *t*-test, *χ*^2^ test, and Fisher’s exact probability test. A value of *p* <0.05 was regarded as statistically significant.

## Results

The clinicopathologic characteristics of informative cases of K-*ras*, mtDNA, and MSI analyses are shown in Tables 1, 2, and 3, respectively. A summary of genetic alterations is shown in Table 4.

**Table 1:** **Clinicopathologic features of informative cases of Vietnamese and Japanese CRCs undergoing K-** ***ras*** **analysis**

	**Vietnamese CRC (n = 24)**	**Japanese CRC (n = 45)**	**p-value**
Age, mean (range)	53.0 (32–79)	62.9 (42–87)	0.006
Sex, male/female	15/9	31/14	0.60
Tumour location			
Right/left side*	6/18	16/29	0.43
Histology			
Well/moderately/poorly differentiated	0/20/4	36/9/0	0.012

*Right-side colon includes the cecum, ascending and transverse colon. Left-side colon includes the descending and sigmoid colon and rectum. CRC, colorectal cancer.

**Table 2:** **Clinicopathologic features of informative cases of Vietnamese and Japanese CRCs undergoing microsatellite instability analysis**

	**Vietnamese CRC (n = 27)**	**Japanese CRC (n = 130)**	**p-value**
Age, mean (range)	50.0 (34–80)	63.2 (32–86)	<0.001
Sex, male/female	18/9	78/52	0.67
Tumour location			
Right/left side*	6/21	35/95	0.79
Histology			
Well/moderately/poorly differentiated	1/23/3	76/54/0	0.005

*Right-side colon includes the cecum, ascending and transverse colon. Left-side colon includes the descending and sigmoid colon and rectum. CRC, colorectal cancer.

**Table 3:** **Clinicopathologic features of informative cases of Vietnamese and Japanese CRCs undergoing mitochondrial DNA analysis**

	**Vietnamese CRC (n = 44)**	**Japanese CRC (n = 133)**	**p-value**
Age, mean (range)	54.0 (32–82)	63.7 (37–88)	<0.001
Sex (male, female)	29/15	82/51	0.61
Tumour location			
Right/left side*	10/34	32/101	0.98
Histology			
Well/moderately/poorly differentiated	1/37/6	41/82/10	0.22

*Right-side colon includes the cecum, ascending and transverse colon. Left-side colon includes the descending and sigmoid colon and rectum. CRC, colorectal cancer.

**Table 4:** **Summary of genetic alterations in Vietnamese and Japanese CRCs**

**Genetic alteration**	**Vietnamese CRCs**	**Japanese CRCs**	**p-value**
K-*ras* mutation, present	8/24 (33%)	5/45 (11%)	0.048
MSI-H, present	6/27 (22%)	10/130 (8%)	0.030
mtDNA mutation, present	19/44 (43%)	11/133 (8%)	<0.001

*CRCs*, colorectal cancers; *MSI-H*, high-frequency microsatellite instability; *mtDNA*, mitochondrial DNA.

**Figure 1:**
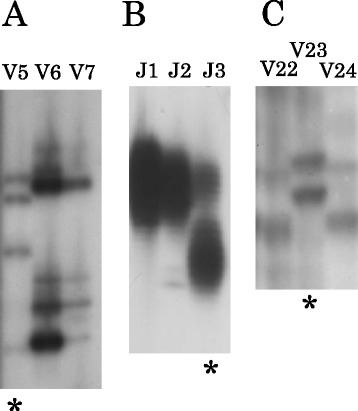
**Representative examples of genetic analyses.** The patient ID number is indicated above each lane. **(A)** K-*ras* mutation by PCR-SSCP analysis. Patient V5 shows mobility shift, indicating the mutation (*). **(B)** High-frequency microsatellite instability (MSI-H) by microsatellite assay. Patient J3 shows additional bands, indicating the MSI-H (*). **(C)** Mitochondrial DNA mutation by microsatellite assay. Patient V23 shows mobility shift, indicating the mutation (*).

K-*ras* mutations were examined in the CRCs of 60 Vietnamese and 45 Japanese patients (Figure 1A). Of these CRCs, 24 from the Vietnamese and all 45 from the Japanese patients were informative in the analysis. The clinicopathologic characteristics of the informative cases are shown in Table 1. The details of the Japanese data have been reported previously [4]. The frequency of the mutations in the Vietnamese CRCs was significantly higher than that in the Japanese CRCs (8 of 24 [33%] vs 5 of 45 [11%], *p* =0.048) (Table 4). There were no significant differences in the clinicopathologic characteristics, such as age, sex, tumour location, and depth, of the Vietnamese and Japanese patients in terms of tumours with or without K-*ras* mutations (data not shown).

MSI-H was examined in the CRCs of 60 Vietnamese and 130 Japanese patients (Figure 1B). Of these CRCs, 27 in the Vietnamese and all 130 in the Japanese patients were informative in the analysis. The clinicopathologic characteristics of the informative cases are shown in Table 2. The frequency of MSI-H in the Vietnamese CRCs was significantly higher than that in the Japanese CRCs (6 of 27 [22%] vs 10 of 130 [8%], *p* =0.030). There were no significant differences in the clinicopathologic characteristics of the Vietnamese and Japanese patients in terms of tumours with or without MSI-H (data not shown).

mtDNA mutations were examined the CRCs of 60 Vietnamese and 138 Japanese patients (Figure 1C). Of these CRCs, 44 in the Vietnamese and 133 in the Japanese were informative in the analysis. The clinicopathologic characteristics of the informative cases are shown in Table 3. The details of the Japanese data have also been reported previously [5]. The frequency of the mutations in the CRCs in the Vietnamese was significantly higher than that in the CRCs of the Japanese (19 of 44 [43%] vs 11 of 133 [9%], *p* <0.001). There were no significant differences in the clinicopathologic characteristics of the Vietnamese and Japanese patients in terms of tumours with or without mtDNA mutations (data not shown).

## Discussion

We demonstrated that the frequencies of mutations of K-*ras* and mtDNA and the frequency of MSI-H were significantly higher in the CRCs of the Vietnamese than in those of the Japanese patients. These results indicate that the developmental pathways of CRCs in the Vietnamese may differ from those of CRCs in the Japanese. This is, to our knowledge, truly the first report of genetic alterations in K-*ras* and mtDNA and of MSI-H in CRCs of Vietnamese patients.

Carcinogenesis and the progression of CRC are multistep processes involving the accumulation of genetic alterations. Much attention has been directed to the study of genetic events such as the activation of oncogenes, inactivation of tumour suppressor genes due to mutations and methylations, defects in mismatch repair genes, and mutations of mtDNA.

K-*ras*, one of the major oncogenes, encodes the 21-kD plasma membrane-bound guanosine triphosphate-binding protein, which is a key regulatory component of signal transduction pathways that transmit growth stimulatory signals from cell surface receptors to intracellular targets [8]. The majority of the mutations involve a single amino acid submission at codon 12 or 13, which decreases the intrinsic guanosine triphosphatase activity and which leads to the constitutive activation of the K-*ras* signaling pathway. K-*ras* mutations are found in 20–30% of CRCs in the Japanese [11,12]. The frequency of K-*ras* mutations in the CRCs of the Japanese in the present study was lower than that in previous reports. This may be due to differences of sensitivity in the assay used and to differences in the examined locus. Even so, K-*ras* mutations were found significantly more frequently in the CRCs of the Vietnamese than Japanese patients. However, there is a significant difference in histology between the CRCs in the Vietnamese and Japanese. Moderately differentiated CRC was predominant (83% of the informative cases) in the Vietnamese, whereas well-differentiated CRC was predominant in the Japanese patients (80% of the informative cases). Although some researchers may think that a difference in histology might be associated with the difference in the frequency of K-*ras* mutations, to date, many reports show no associations between K-*ras* mutations and histologic grades [13-15], and therefore, other factors may be associated with the higher frequency of K-*ras* mutations in the Vietnamese CRCs. It is well known that K-*ras* mutations are associated with polypoid-type colorectal tumours but not with flat-type tumours [16]. The data on macroscopic subtypes in the CRCs examined in the present study were insufficient because most of the CRCs were at advanced stages; thus, it was unclear whether they were of polypoid- or flat-type origin. The association of K-*ras* mutations and macroscopic views in the Vietnamese CRCs should be examined in the early stages of tumours. If the polypoid type, which contains a high frequency of K-*ras* mutations, is predominant in these CRCs, this may be another characteristic unique to Vietnamese CRCs.

Recently, mutations of mtDNA have been reported in various types of human cancers [5,17], as have frequent mutations of the D-loop region of mtDNA [18-20]. The D-loop is the region that is critical for replication and expression of the mitochondrial genome because it contains the leading-strand origin of replication and the major promoters for transcription. Habano et al. [21] reported that 44% of Japanese CRCs have mutations of the D-loop region. The frequency of mtDNA mutations in the Japanese CRCs in the present study was lower than that reported by Habano et al. This may be due to differences in the sensitivity of the assay used and to differences in the examined locus. Regardless, the frequency of the mutations in the CRCs of the Vietnamese was significantly higher than that in the CRCs of the Japanese patients. mtDNA mutations are also reported to have no association with histologic differences or K-*ras* mutations in many studies [20].

MSI-H is caused by the inactivation of DNA mismatch repair genes such as *hMSH2* and *hMLH1* [6]. Approximately 90% of CRCs in patients with hereditary non-polyposis CRC (HNPCC) show MSI-H. MSI-H CRCs have been reported to be more frequently associated with early onset, proximal tumour location, poorly differentiated and mucin-containing histology, low lymph node metastasis, and better survival rate [20]. MSI-H has been detected in 6–14% of Japanese CRCs [22,23], and our result was similar to that in these previous reports. In the present study, the frequency of MSI-H was significantly higher in the Vietnamese CRCs than in the Japanese CRCs. It is hypothesised that HNPCC may occur more frequently in the Vietnamese than in the Japanese. The incidence of early-onset CRC in Vietnamese has been reported to be high [2]. The finding that a higher frequency of poorly differentiated histology was observed in the Vietnamese CRCs than in the Japanese CRCs in the present study is compatible with this hypothesis. Further studies to clarify the frequency of HNPCC in the Vietnamese population are needed.

Why is the incidence of early-onset CRC in the Vietnamese greater than that in the Japanese, and why do Vietnamese CRCs contain higher frequencies of genetic alterations, especially those of K-*ras* and mtDNA mutations? One possible reason might be the differences in ethnicity between the two populations. A second possible reason might be the differences in cultural habits including diet and lifestyle between the two peoples. Although both countries are in Asia, Vietnam is a developing country, and Japan is a developed country. Common diseases differ between the countries: infectious diseases are prevalent in Vietnam, whereas lifestyle-related diseases dominate in Japan. Differences in environmental factors may also be associated with the differences in the genetic background of the CRCs. A third possible reason might be found in the history of Vietnam. During the Vietnamese war, American military forces spread great amounts of the herbicide Agent Orange in Vietnam. The agent was a mixture of 2,4-dichlorophenoxyacetic acid (2,4-D) and 2,4,5-trichlorophenoxyacetic acid (2,4,5-T), which was contaminated with dioxin, a toxic chemical shown to have carcinogenic effects [24]. Among US military veterans who were in the Vietnamese war, the risk of cancer at any site including CRC is increased [25].

There are several limitations in the present study. First is the relatively small number of patients included, for which sampling bias might exist. All of the Vietnamese samples were obtained in a university medical centre in Ho Chi Minh, and it is possible that the patients were not a good representation of the whole Vietnamese population. Likewise, all of the Japanese samples were obtained in a university hospital in Hiroshima, and these patients also may not be representative of the whole Japanese population. Second is the number of genes examined; we examined only K-*ras*, mtDNA, and MSI-H. A more detailed view is needed. Third is the presence of a possible bias in the sampling period of the two groups. Differences in diagnosis might exist between the relatively small sampling period of 2 years in the Vietnamese and 12 years in the Japanese. In addition, an increase in the frequency of K-*ras* mutations in CRCs in the 1990s compared with that in the 1960s has been reported [26]. This implies that the frequency of genetic alterations may differ during different time periods.

## Conclusions

We demonstrated that CRCs in the Vietnamese patients may involve significantly more frequent changes in K-*ras* and mtDNA genes and in microsatellite regions compared with CRCs in the Japanese patients, indicating that the developmental pathways of CRCs in the Vietnamese may differ from those in the Japanese. Further studies with a larger number of patients and more target genes are needed to clarify the differences between CRCs in the Vietnamese and Japanese populations.
